# Helminth infections and type 2 diabetes: a cluster-randomized placebo controlled SUGARSPIN trial in Nangapanda, Flores, Indonesia

**DOI:** 10.1186/s12879-015-0873-4

**Published:** 2015-03-18

**Authors:** Dicky L Tahapary, Karin de Ruiter, Ivonne Martin, Lisette van Lieshout, Bruno Guigas, Pradana Soewondo, Yenny Djuardi, Aprilianto E Wiria, Oleg A Mayboroda, Jeanine J Houwing-Duistermaat, Hengki Tasman, Erliyani Sartono, Maria Yazdanbakhsh, Johannes W A Smit, Taniawati Supali

**Affiliations:** Department of Internal Medicine, Faculty of Medicine, Universitas Indonesia, Jakarta, Indonesia; Department of Parasitology, Leiden University Medical Center, Leiden, The Netherlands; Department of Medical Statistics and Bioinformatics, Leiden University Medical Center, Leiden, The Netherlands; Department of Mathematics, Parahyangan Catholic University, Bandung, Indonesia; Department of Molecular Cell Biology, Leiden University Medical Center, Leiden, The Netherlands; Department of Parasitology, Faculty of Medicine, Universitas Indonesia, Jakarta, Indonesia; Center for Proteomics and Metabolomics, Leiden University Medical Center, Leiden, The Netherlands; Department of Mathematics, Faculty of Mathematics and Natural Science, Universitas Indonesia, Jakarta, Indonesia; Department of Internal Medicine, Radboud University Medical Centre, Nijmegen, The Netherlands; Department of Internal Medicine, Leiden University Medical Center, Leiden, The Netherlands

**Keywords:** Insulin resistance, Helminth, Type 2 diabetes, Parasite, Metabolism, Albendazole, Immunology

## Abstract

**Background:**

Insulin resistance is a strong predictor of the development of type 2 diabetes mellitus. Chronic helminth infections might protect against insulin resistance via a caloric restriction state and indirectly via T-helper-2 polarization of the immune system. Therefore the elimination of helminths might remove this beneficial effect on insulin resistance.

**Methods/Design:**

To determine whether soil-transmitted helminth infections are associated with a better whole-body insulin sensitivity and whether this protection is reversible by anthelmintic treatment, a household-based cluster-randomized, double blind, placebo-controlled trial was conducted in the area of Nangapanda on Flores Island, Indonesia, an area endemic for soil-transmitted helminth infections. The trial incorporates three monthly treatment with albendazole or matching placebo for one year, whereby each treatment round consists of three consecutive days of supervised drug intake. The presence of soil-transmitted helminths will be evaluated in faeces using microscopy and/or PCR. The primary outcome of the study will be changes in insulin resistance as assessed by HOMA-IR, while the secondary outcomes will be changes in body mass index, waist circumference, fasting blood glucose, 2 h-glucose levels after oral glucose tolerance test, HbA1c, serum lipid levels, immunological parameters, and efficacy of anthelmintic treatment.

**Discussion:**

The study will provide data on the effect of helminth infections on insulin resistance. It will assess the relationship between helminth infection status and immune responses as well as metabolic parameters, allowing the establishment of a link between inflammation and whole-body metabolic homeostasis. In addition, it will give information on anthelmintic treatment efficacy and effectiveness.

**Trial registration:**

This study has been approved by the ethical committee of Faculty of Medicine Universitas Indonesia (ref: 549/H2.F1/ETIK/2013), and has been filed by the ethics committee of Leiden University Medical Center, clinical trial number: ISRCTN75636394. The study is reported in accordance with the CONSORT guidelines for cluster-randomised trials.

## Background

The number of people with diabetes mellitus is increasing worldwide [1-3]. At present, 8.3% of adults (382 million people) have diabetes mellitus [4] and Asia is a major site of this rapidly emerging epidemic [5]. In many Asian countries, including Indonesia, rapid socio-economic development has led to a shift in infrastructure, technology and introduction of Western style diets, which promotes overnutrition and sedentary lifestyles [5-8]. These changes have already led to an increasing prevalence of diabetes mellitus in Indonesia [9-12].

A strong predictor for the development of type 2 diabetes mellitus (DM2) is insulin resistance [13,14], which is caused by complex disturbances in multiple biological systems. There is now abundant evidence that inflammation [15] plays a role in the development of DM2, in addition to the more established relationship between an altered energy balance resulting from excess consumption of high-energy foods and/or decreased physical activity. In DM2 subjects, chronic low-grade inflammation is a common feature [15] which results, at least in part, from the activation of inflammatory pathways by fatty acids in multiple organs [16-18]. However, the fundamental molecular mechanisms are still incompletely understood [19].

In developing countries, infectious pressure might be one particular modifier of insulin resistance. Helminth infections, which are still endemic in many low to middle income countries, may therefore affect whole-body and tissue-specific insulin sensitivity owing to their immunomodulatory properties [20]. Previous studies have shown that helminth infections can adopt an immune evasion strategy by inducing regulatory T cells [21-26]. Hereby helminth infections may decrease systemic inflammation and subsequently the development of inflammatory diseases, including DM2 [27-30]. Studies examining the relationship between helminth infections and DM2 in both humans [31,32] and murine models [33,34] support this hypothesis. At a molecular level, mTOR, a serine/threonine protein kinase located downstream of insulin signalling, plays an essential role in immune cell energy metabolism and function [35,36]. Furthermore, it has been shown that STAT6 signalling downstream of IL-4, as well as Th2 responses induced by helminths, improve glucose metabolism and insulin signalling [33,37]. Intriguingly, in humans, immune intervention with IL-1 receptor antagonist (Anakinra) has also been shown to influence glucose metabolism [38].

Helminths are also known to reduce energy intake and thereby change the energy balance [39], which may be beneficial in terms of insulin resistance [39,40]. Helminths may therefore both directly improve insulin sensitivity via a caloric restriction state and indirectly via Th2 activation. It appears, the immune system which has evolved with helminths [41] and under conditions of low energy intake, seems to be out of balance in situations of nutritional overload and decreasing exposure to parasites [23,42]. In line with the proposed beneficial effects of helminth infections on glucose metabolism, our previous unpublished cross sectional study in Flores Island, Indonesia, has shown that subjects infected with intestinal helminths have a significantly lower insulin resistance as expressed by HOMA-IR.

Although aforementioned studies strongly suggest that there is an association between helminth infections, systemic inflammation and glucose metabolism, the causality in these relationships has not been demonstrated as yet. Therefore we have initiated a large scale cluster randomized controlled trial (RCT) with the aim to assess the effect of anthelmintic treatment on insulin resistance, the hypothesis being that reduction of worm load to undetectable levels will lead to a higher degree of insulin resistance. While study outcomes will be analysed at the individual participant level, a household cluster randomization was chosen to minimise contamination between treatment groups and therefore reinfection of treated individuals.

## Study design

### Study area

The study area is located in Nangapanda, a sub-district of the Ende District of Flores Island, Indonesia [43,44]. Nangapanda is a semi-urban coastal area with a population of approximately 22.000 people being divided over 29 villages. Our study area includes three of these villages (Ndeturea, Ndorurea 1, Ndorurea), with a total population of 3698 people, from which most of the adult population are farmers. Previous studies have shown that this area is endemic for soil-transmitted helminth (STH) infections [45]. A detailed map of the study area has been published [43].

### Trial design

The study is designed as a household-based cluster-randomized, double-blind trial with two arms. In one arm treatment is given with albendazole (single dose of 400 mg) on 3 consecutive days, while the other arm consists of matching placebo treatment (both albendazole and placebo are manufactured by PT Indofarma Pharmaceutical, Bandung, Indonesia). The treatment is provided every three months for a period of 1 year (total 4 rounds) to all household members except children below 2 years of age, while subjects aged 16 and above will undergo clinical and laboratory examination. Subjects with active treatment for diabetes mellitus, serious concomitant disease and pregnancy will be excluded.

The population was randomised by JWAS and JJH using computer aided block randomization at household level, utilizing Random Allocation Software to assign treatment groups. Both study investigators and patients are blinded for treatment codes. The treatment code will be unblinded when all data needed for analysis are cleaned and entered into the database. An additional randomization was performed in a subgroup of individuals, who will undergo an oral glucose tolerance test and immunological studies in order to study glucose metabolism and immune mechanisms in more detail. For this subgroup, we aimed to select one subject per household and stratified by age group (16–36 years of age, 36–56 years of age, and >56 years of age) to ensure that sufficient numbers of all age groups are participating. Randomization was based on households.

Well trained community workers were recruited and trained to distribute the drugs. These workers were also trained to assist during clinical examination and sample collection and were involved in health promotion within the population. Community workers and research team members will directly supervise the study participants while taking the study medication, and will collect empty drug canisters at each visit to confirm compliance. Furthermore, assessment of side effects will take place during these visits and migration and death will be noted. Adverse events spontaneously reported by the patient or observed by the investigators, will be monitored throughout the study. After completion of the study, the whole study population will be treated with a single dose of albendazole (400 mg) for 3 consecutive days.

### Outcomes

As this study aims to assess the effect of anthelmintic treatment on whole-body insulin sensitivity, our primary outcome is a change in insulin resistance as assessed by HOMA-IR between both treatment arms after one year of treatment. Secondary outcomes are changes in body mass index and waist circumference, fasting blood glucose, 2 h-glucose levels after oral glucose tolerance test, HbA1c, serum lipid levels, immunological parameters, and efficacy of anthelmintic treatment.

### Sample size

Sample size is calculated according to intention to treat analysis in which we will need 1580 subjects in total. Based on our previous study [45] we assume that the average household size is 4 and that around 20% will be lost to follow up after one year. We use a significance level of 5% and a power of 80%. Correlations within households are taken into account by using the correction factor 1 + (m-1) ICC, with m being the household size and ICC the intra-class correlation. The sample size is computed for a difference in mean between the two treatment groups of 0.18 and an ICC of 0.1.

For the subgroup of individuals undergoing an oral glucose tolerance test, a sample size of 335 subjects in total is calculated assuming that around 20% will be lost to follow up after one year and using a significance level of 5% and a power of 80%. The sample size is computed for a difference in mean of 10.3 mg/dL and a standard deviation of glucose level of 30 mg/dL.

## Methods

### Sample collection

At baseline all eligible subjects, aged 16 and above will be invited to visit the examination centre after an overnight fasting and to provide stool, blood and first morning urine samples. During this visit, participant’s education level and profession will be registered. After 1 year of treatment follow-up sample collection will take place as shown in Table 1.

**Table 1:** **Study schedule of the Sugarspin project**

**Outcome**	**Baseline**	**3 monthly treatment**				**1 year follow up**
		**1st**	**2nd**	**3rd**	**4th**	
Clinical Anthropometry	**X**					**X**
Parasitological examination	**X**					**X**
Metabolic parameters	**X**					**X**
Immunological parameters	**X**					**X**
Assessment of side effects		**X**	**X**	**X**	**X**	**X**

### Clinical anthropometry assessment

Anthropometric measurements of body weight, height, waist and hip circumference are obtained using the National Heart, Lung, and Blood Institute (NHLBI) practical guidelines. To measure body weight a flat scale for mobile use (SECA Model 876, Seca Gmbh Co, Hamburg, Germany) is used, while a portable stadiometer (SECA Model 213, Seca Gmbh Co, Hamburg, Germany) is used to measure height. Waist and hip circumference are measured using ergonomic circumference measuring tape (SECA Model 201, Seca Gmbh Co, Hamburg, Germany). In addition, body fat composition is measured using a Tanita body composition analyser (TBF-300A, Tanita Corp, Tokyo, Japan). Three blood pressure measurements (left arm, sitting upright position, after resting 5 minutes) are taken from each subject, using a digital sphygmomanometer (HEM-7200, Omron Healthcare Co, Ltd, Kyoto, Japan), and calibrated using a Riester nova-presameterH-Desk model mercury sphygmomanometer (Gerhard Glufke Rudolf Riester GmbH & Co, Jungingen, Germany) and a 3MTM LittmannH Classic II S.E. Stethoscope (3 M, St. Paul, Minnesota, USA). The average of three systolic/diastolic blood pressure measurements will be used for analysis.

### Parasitological examination

To assess intestinal helminth infection, stool containers are distributed and collected by health workers. Stool samples are examined by the Kato Katz method [46] for identification and quantification of STH eggs using 2 slides for each sample. An aliquot of the fresh stool samples is frozen at −20°C in the field and subsequently at −80°C in laboratories of the Departments of Parasitology at Leiden University Medical Center, Leiden, The Netherlands and Faculty of Medicine Universitas Indonesia, Jakarta, Indonesia for DNA extraction [43]. Part of the stool sample will be saved for potential future analysis of the microbiome.

#### DNA isolation and helminth real-time PCR

DNA isolation from stool will be performed as described elsewhere [43], with some minor modifications. Real-time PCR will be performed to detect the presence of *A. duodenale*, *N. americanus* (hookworm), *A. lumbricoides* and *T. trichiura* using a method described previously [43] with some modifications.

### Blood collection

Peripheral blood is collected into EDTA and SST Vacutainers (BD, Franklin Lakes, NJ, USA). Giemsa-stained peripheral blood smear is prepared to evaluate neutrophil and eosinophil counts. In a subset of the study population, additional blood is collected in PAXgene Blood RNA Tubes (PreAnalytiX GmbH, Hombrechtikon, Switzerland) and Sodium Heparin Vacutainers (BD). Blood collected in PAXgene Blood RNA Tubes will be used to study RNA expression profiles, while blood collected in Sodium Heparin Vacutainers will be used for detailed immunological measurements as described below (section Immunological methods). All samples deriving from EDTA and SST Vacutainers (serum, plasma, cell pellet and whole blood) and all PAXgene Blood RNA Tubes are kept at −20°C at the Field Clinical Research Centre (FCRC) and will be sent on dry ice to the University’s laboratory for storage at −80°C.

### Metabolic parameters

Fasting blood glucose is measured in capillary blood using Breeze®2 glucose meters (Bayer Health Care LLC, Basel, Switzerland). An oral glucose tolerance test is performed in a subset of the study population according to the WHO protocol [47,48]. Glucose levels are measured in capillary blood using Breeze®2 glucose meters after overnight fasting and 2 hours after ingesting 75 g of anhydrous glucose dissolved in 200 cc of water. Insulin, HbA1c and lipid profiles will be measured in an internationally accredited laboratory. HOMA-IR, a well-validated measure of insulin resistance will be calculated to estimate insulin resistance [49].

### Immunological methods

The immunological parameters that will be studied are 1) Total IgE levels as one of the markers of a Th2 response and its relation to metabolic parameters, 2) Circulating pro- and anti-inflammatory cytokines in order to study their relationship to metabolic parameters, 3) Antigen specific IgE and IgG to *Ascaris lumbricoides* to monitor antibody responses to one of the helminths studied as a marker of changing immune responses as a result of anthelmintic treatment, 4) Granulocyte (neutrophil and eosinophil) frequencies and their activation to assess whether granulocytes, in particular eosinophils which are associated with Th2 response, are linked to helminth infections and metabolic parameters, 5) Peripheral blood mononuclear cells (PBMC) subset analysis and polarisation by flow cytometry in order to assess the relationship between immune cell frequencies (T cell subsets, B cell subsets, monocyte subsets, NK cells and myeloid suppressor cells) in situ as well as after activation and metabolic parameters.

#### Total IgE

Total IgE will be measured using ELISA with rabbit anti-human IgE antibodies (Dako, Glostrup, Denmark) and goat anti-human IgE biotinylated antibodies (Vector Laboratories, Burlingame, CA, USA) as capture and detection antibodies, as described previously [43]. The World Health Organization standard of human serum IgE was used as a reference (National Institute for Biological Standards and Control). The results will be expressed in International Units (IU).

#### Circulating cytokines

Pro and anti-inflammatory cytokines (TNFα, IFNγ, IL-1, IL-6, IL-10, TGFβ) will be measured in serum samples using cytokine kits with high sensitivity.

#### Ascaris-specific IgE

*Ascaris* antigen will be prepared from *Ascaris lumbricoides* worms as described previously [50]. Maxisorp plates (Thermo Fisher Scientific, Roskilde, Denmark) will be coated overnight with 5 μg/ml *Ascaris* antigen in 0.1 M carbonate buffer (pH 9.6). Plates will be blocked for 1 hour with PBS containing 2% bovine serum albumin. Samples will be diluted 1/60 in 0.1 M Tris–HCl containing 0.05% Tween-20 and incubated overnight together with a pool of positive standard plasma containing 1 × 10^6^ arbitrary units (AU) parasite specific IgE. After a washing step, goat anti-human IgE biotinylated antibodies (Vector) will be incubated followed by streptavidin-HRP (Sanquin, Amsterdam, the Netherlands). The color is developed by adding 3,3′,5,5′ tetramethylbenzidine (TMB) (KPL, Gaithersburg, MD, USA). The reaction will be stopped by adding 1.8 M H_2_SO_4_ and absorbance will be read at 450 nm in an automated plate reader.

#### Ascaris-specific IgG isotypes

Maxisorp plates will be coated with *Ascaris* antigen as described for *Ascaris* specific IgE above. Blocking will be done using PBS containing 5% bovine serum albumin. Samples will be diluted 1/1000, 1/10, 1/5 or 1/25 for IgG1, IgG2, IgG3 and IgG4 respectively, and a pool of positive standard plasma containing 1 × 10^6^ arbitrary units (AU) parasite specific IgG isotypes will be included in each plate. After overnight incubation, HRP-labelled anti human IgG isotypes (Sanquin) in PBS 0.05% Tween-20 will be added for 4 hours incubation at 37°C using the following dilutions: 1/3000 for anti IgG1 (HP6188) and anti IgG4 (HP6196); 1/1000 for anti IgG2 (HP6014) and anti IgG3 (HP6095). TMB substrate will be used to develop the color and the reaction will be stopped as described above.

#### Whole blood stimulation and fixed granulocyte cryopreservation

To study the expression of activation markers on granulocytes, 600 μl of heparinised venous blood is divided over 3 polystyrene tubes (200 μl/tube). After a pre-incubation of 5 minutes in a 37°C waterbath, a 5 minutes stimulation at 37°C is performed with N-Formyl-Met-Leu-Phe (FMLP, 10^−5^ M; Sigma, Saint Louis, MO, USA) or eotaxin (10^−7^ M; R&D systems, Abingdon, UK). Subsequently, 4 ml of FACS lysing solution (BD) is added and after an incubation period of 15 minutes at room temperature the red blood cells is lysed while white blood cells, including granulocytes, become fixed. Cells are washed with RPMI 1640 containing 10% heat-inactivated FCS and then resuspended in RPMI 1640 containing 10% of heat-inactivated foetal calf serum (FCS) and 10% dimethyl sulfoxyde (DMSO). Cryovials containing the cell suspension are placed at −80°C for minimum of 4 hours, followed by storage in liquid nitrogen until analysis.

#### PBMC cryopreservation

Peripheral blood mononuclear cells (PBMCs) are isolated from heparinised venous blood using Ficoll density gradient centrifugation within 12 hours after blood collection. After isolation, cells are cryopreserved in RPMI 1640 containing 20% of heat-inactivated foetal calf serum (FCS) and 10% dimethyl sulfoxyde (DMSO). Cryovials containing the cell suspension are transferred to a freezing unit which is placed in a −80°C freezer for minimum of 4 hours. Subsequently, vials are stored in liquid nitrogen until analysis.

### Metabolomics for metabolic profiling

Urine samples and blood samples from heparinized blood are kept at −20°C at the FCRC and subsequently stored at −80°C at the University's laboratory for future metabolomics measurements. The exploratory metabolomics analysis will be performed by 1H-NMR and LC-MS metabolomics, a combination of NMR and LC-MS proposed for this study provides a comprehensive coverage of metabolome and as such increase the probability of finding physiologically meaningful associations within the data.

### Data management and statistical analyses

A centrally accessible database designed in Microsoft Access is established and the data is entered by well-trained data entry personnel. Descriptive data will be summarized for continuous variables as mean +/− SD for normally distributed data and median (range) for non-normally distributed data. Categorical data will be expressed as proportions.

The effect of anthelmintic treatment on insulin resistance (HOMA-IR) as our primary outcome will be assessed using an intention to treat approach after 1 year of treatment using mixed models to account for the correlation within households, in which relevant confounders (including age, gender, BMI, village) are entered. The characterization of immune responses to helminth infections and systemic inflammation will be assessed by measuring cytokine profiles. Moreover, for these analyses multilevel modelling will be used and the use of longitudinal data will take repeated measurement into account [51]. The mediation of helminth’s effects on insulin resistance via immune responses will also be assessed.

### Ethical approval, trial registration and consent

This study has been approved by The Health Research Ethical Committee, Faculty of Medicine, Universitas Indonesia Cipto Mangunkusumo Hospital, Jakarta, Indonesia (reference number:549/H2.F1/ETIK/2013). It has also been filed by the ethics committee of Leiden University Medical Center and is registered as a clinical trial ref: ISRCTN75636394 (http://www.isrctn.com/ISRCTN75636394). The local health authorities have been informed about this study and have given their approval and support. The study, its benefits and risks are explained to the population and consent forms are distributed to be signed by the subjects who are willing to participate in this study. They are informed that they can withdraw from the study at any time, for any reasons and without any consequences.

### Description of the population recruited

**Figure 1:**
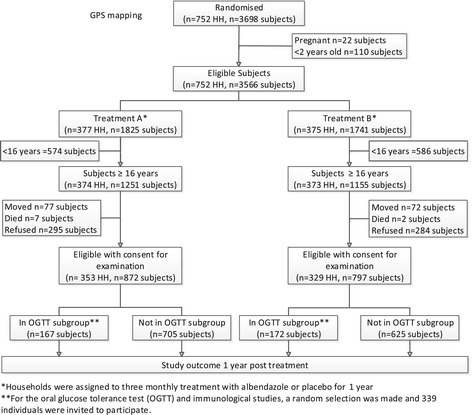
**Flow diagram of the Sugarspin project.**

So far, the study has provided the following data (Figure 1). At baseline, a total of 3698 individuals were registered in 752 households. Of the 2428 subjects aged 16 years or older, 1669 subjects were eligible with consent for examination. For the oral glucose tolerance test and immunological studies 339 subjects were randomly selected and gave approval.

**Figure 2:**
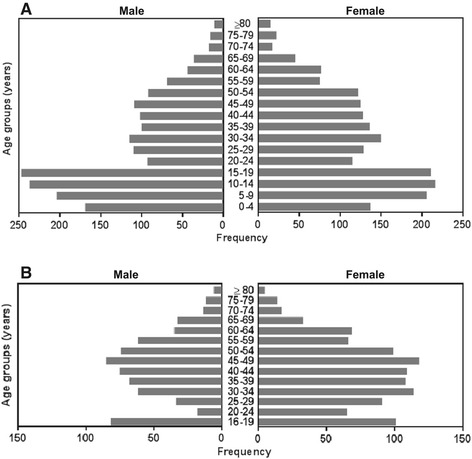
**Age pyramid.** Age pyramid of all individuals living in the study area in Nangapanda, Flores Island, Indonesia (n = 3698 subjects, 52% female) **(A)**, and of study participants (n = 1669 subjects, 60% female) **(B)**.

**Figure 3:**
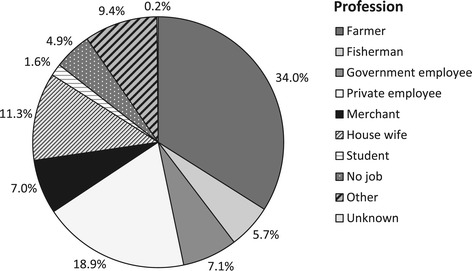
**Job distribution.** At baseline, profession was assessed for study participants (n = 1669 subjects).

**Figure 4:**
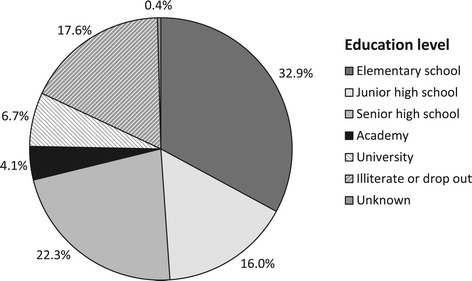
**Education level.** At baseline, education level was assessed for study participants (n = 1669 subjects).

Figure 2 shows the age pyramid of both the total population in the study area and the study population. In the study population farming and fishing are the traditional source of income while some individuals engage in jobs at government offices or in the private sector (Figure 3), a similar distribution is seen in the total population. The education level of the majority of subjects in the study population is elementary school (33%), followed by senior high school (22%), and junior high school (16%), while 11% has college or university degrees. Moreover, 18% of the subjects is illiterate, either not educated at all or dropped out from elementary school (Figure 4). A similar distribution of the education level is seen in the total population.

## Discussion

The SUGARSPIN trial is the first and currently the only longitudinal study investigating the effects of anthelmintic treatment on whole-body insulin sensitivity. This placebo-controlled trial enables us for the first time to investigate the causal relationship between helminth infections, systemic inflammation and glucose metabolism in humans. In addition, this study will provide data on anthelmintic treatment efficacy and effectiveness in a large population in a developing country like Indonesia.

## Abbreviations

DM2: Type 2 diabetes mellitus
FCRC: Field clinical research center
OGTT: Oral glucose tolerance test
STH: Soil-transmitted helminth
Th2: T-helper-2
